# Supplementary material for: HBO1 catalyzes lysine lactylation and mediates histone H3K9la to regulate gene transcription
Source: Nat Commun. 2024 Apr 26;15:3561. doi: 10.1038/s41467-024-47900-6 (PMC11053077; doi:10.1038/s41467-024-47900-6)
Supplement: Supplementary file 1 — Supplementary Information [file 41467_2024_47900_MOESM1_ESM.pdf]

# Supplementary Information

# HBO1 catalyzes lysine lactylation and mediates histone H3K9la to regulate gene transcription

Ziping Niu,[1, #] Chen Chen,[1, #,*] Siyu Wang, [1, #] Congcong Lu,[2, #] Zhiyue Wu,[1] Aiyuan Wang,[1] Jing Mo,[3] Jianji Zhang,[1] Yanpu Han,[1] Ye Yuan,[4] Yingao Zhang,[1] Yong Zang,[1] Chaoran He,[1] Xue Bai,[1] Shanshan Tian,[1] Guijin Zhai,[1] Xudong Wu,[4] and Kai Zhang[1,5,*]

[1] The Province and Ministry Co-sponsored Collaborative Innovation Center for Medical Epigenetics, Key Laboratory of Immune Microenvironment and Disease (Ministry of Education), Tianjin Key Laboratory of Medical Epigenetics, Department of Biochemistry and Molecular Biology, School of Basic Medical Sciences, Tianjin Medical University, Tianjin 300070, China.

[2] Frontiers Science Center for Cell Responses, College of Life Sciences, Nankai University, Tianjin 300071, China.

[3] Department of Pathology, Tianjin Medical University, Tianjin, 300070, China.

[4] Department of Cell Biology, School of Basic Medical Sciences, Tianjin Medical University, Tianjin 300070, China.

[5] Tianjin Key Laboratory of Retinal Functions and Diseases, Eye Institute and School of Optometry, Tianjin Medical University Eye Hospital, Tianjin Medical University, Tianjin 300070, China.

[#] These authors contributed equally to this work.
* Correspondence: K.Z., kzhang@tmu.edu.cn and C.C., chench@tmu.edu.cn

## Supplementary Figures

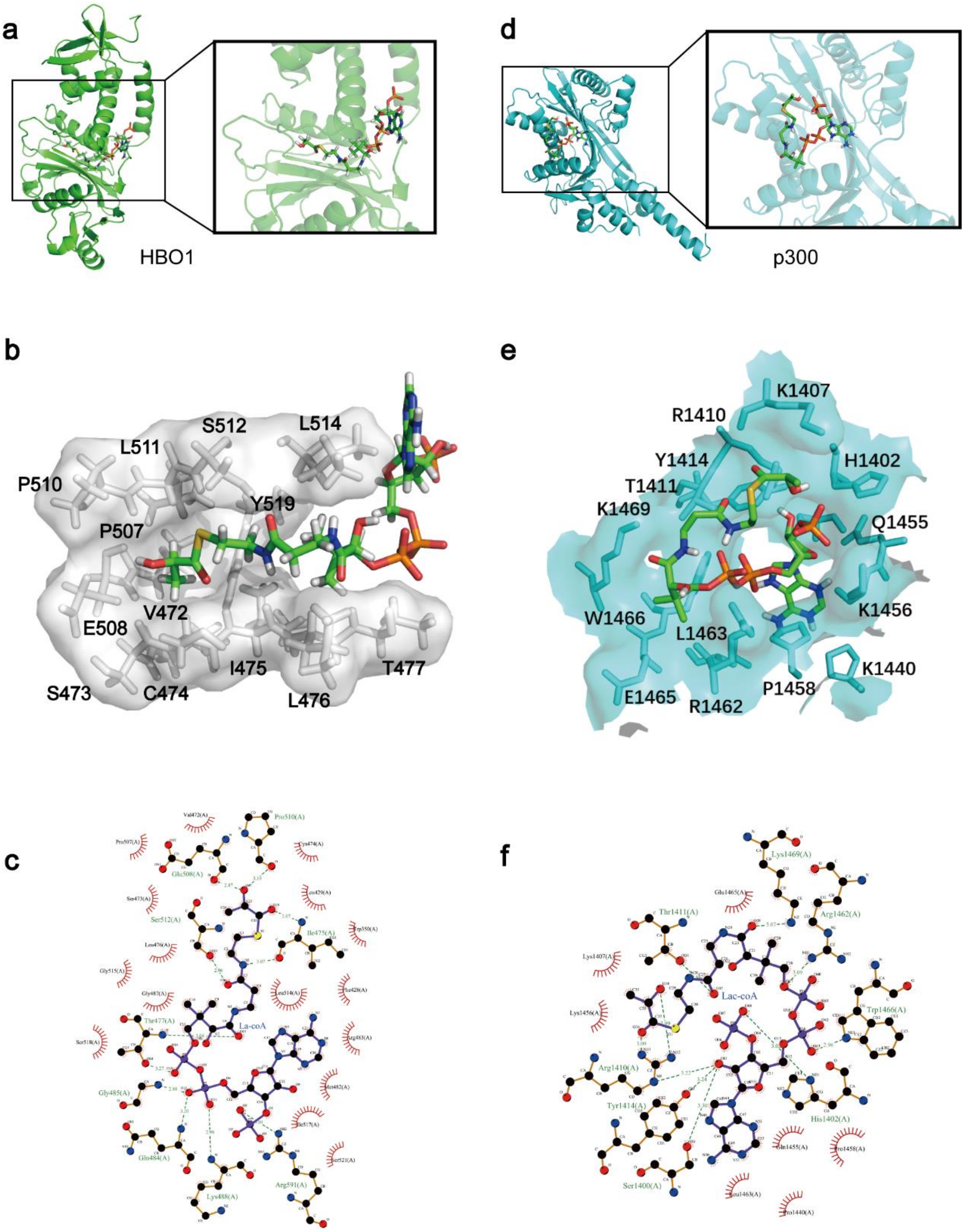


**Supplementary Fig. 1. Comparison of molecular docking of HBO1 and p300 with lactyl-CoA. a-c** Molecular docking simulation of HBO1 binding to lactyl-CoA. In the overall structure diagram **(a)**, HBO1 is represented by ribbons and lactyl-CoA is represented by ball-and-stick structures. In the spatial filling model **(b)**, the ball-and-stick structure represents lactyl-CoA. LigPlot **(c)** showed

the distribution of amino acids in contact with HBO1 and acetyl-coA, with dashed lines representing hydrogen bonds and red arcs representing hydrophobic interactions. **d-f** Molecular docking simulation of p300 binding to lactyl-CoA. In the overall structure diagram **(d)**, p300 is represented by ribbons and lactyl-CoA is represented by ball-and-stick structures. In the spatial filling model **(e)**, the ball-and-stick structure represents lactyl-CoA. LigPlot **(f)** showed the distribution of amino acids in contact with p300 and acetyl-coA, with dashed lines representing hydrogen bonds and red arcs representing hydrophobic interactions.

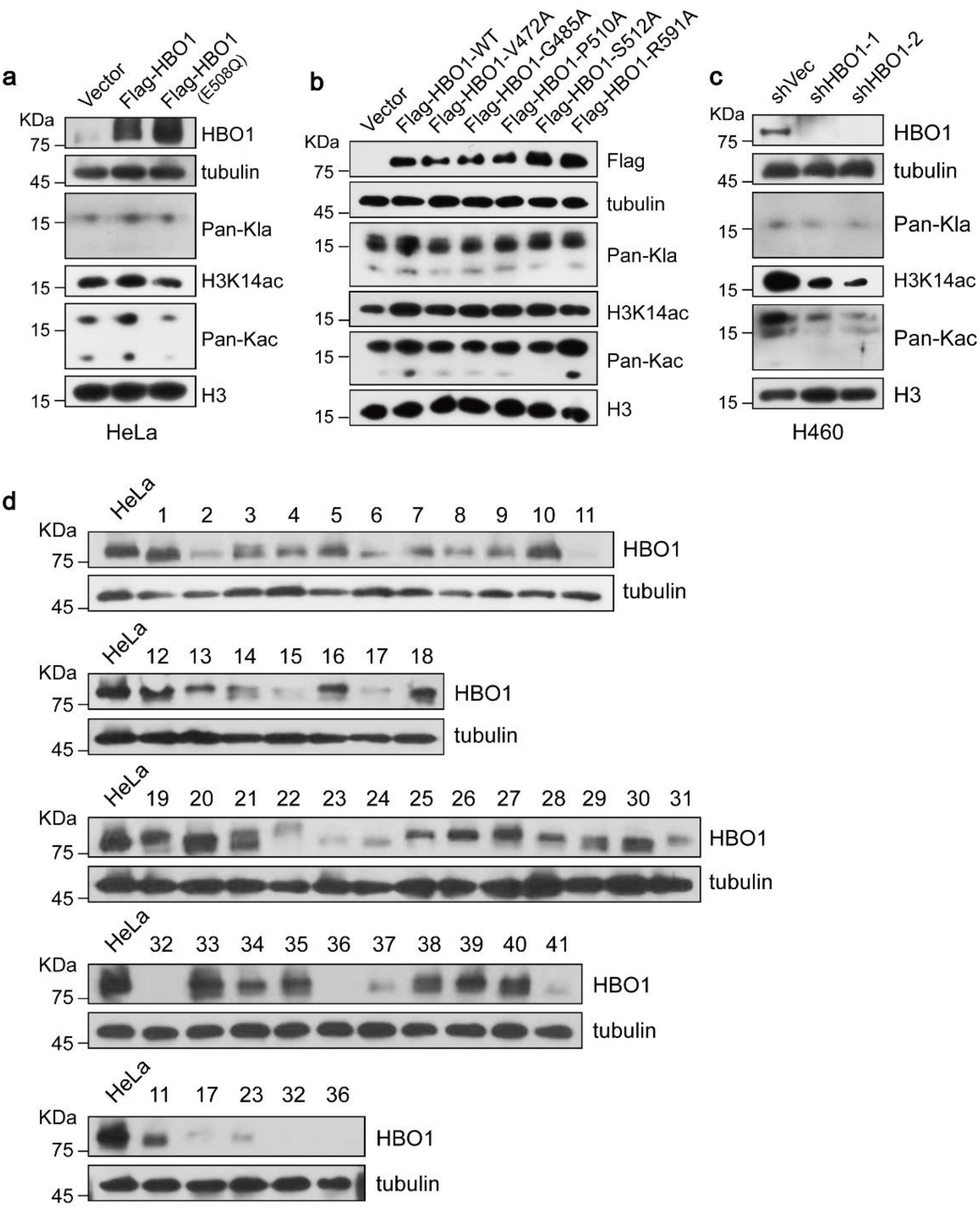


**Supplementary Fig. 2. HBO1 exerts its lactylation catalytic activity through key amino acid residues and screening of HeLa-HBO1-KO clonal cells. a** Western blotting analysis showed overexpression of HBO1 but not HBO1 (E508Q) in HeLa cells increased histone lactylation modification levels, and the vector was used as control. **b** Western blotting analysis showed screening the effect of overexpression of HBO1 and its mutants on the level of histone lactylation modification. **c** Western blotting analysis showed transient HBO1 knockdown by lentiviral-based shRNAs leads to downregulation of histone lactylation modification in H460 cells. **d** Western blotting analysis showed the screening process of two isolated clonal cells. 41 clonal cells were screened from HBO1-KO mixed clones and divided into four groups for western blotting analysis, and the most obvious 1 to 2 clonal cells were selected from each group for further detection. Among the 5 selected clonal cells, the most obvious number 32 and 36 were selected and named as HBO1-KO-1 and HBO1-KO-2 for follow-up experiments. All immunoblots had three biological repetitions, with similar results. Source data are provided as a Source Data file.

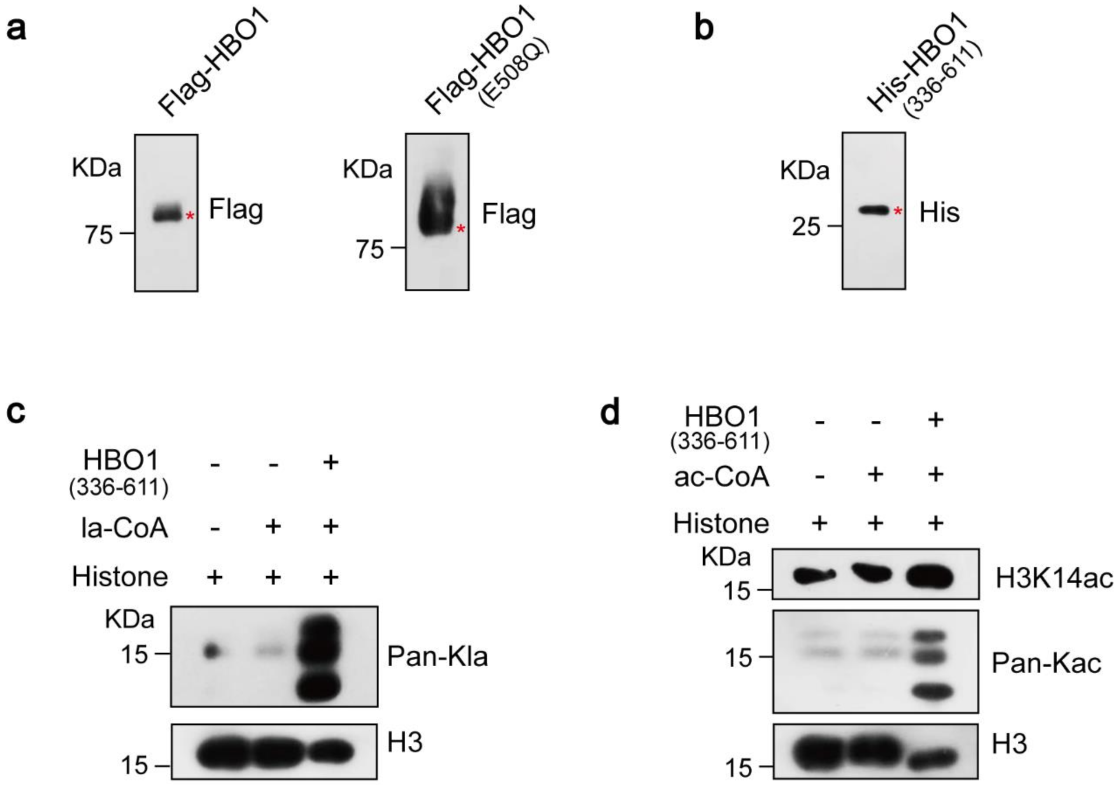


**Supplementary Fig. 3. HBO1 has lactylation catalytic activity for histone in vitro. a** Flag-HBO1 and Flag-HBO1 (E508Q) were expressed in HEK293T cells and the target protein was purified by immunoaffinity. **b** His-HBO1 (336-611) was expressed in *E. coil* BL21 (DE3) and purified with HisPur Ni-NTA Resin. **c-d** HBO1 (336-611) obviously increased histone lactylation levels in vitro. HBO1 (336-611) was incubated with lactyl-CoA **(c)** or acetyl-CoA **(d)** respectively for histone acylation assay in vitro. All immunoblots had three biological repetitions, with similar results. Source data are provided as a Source Data file.

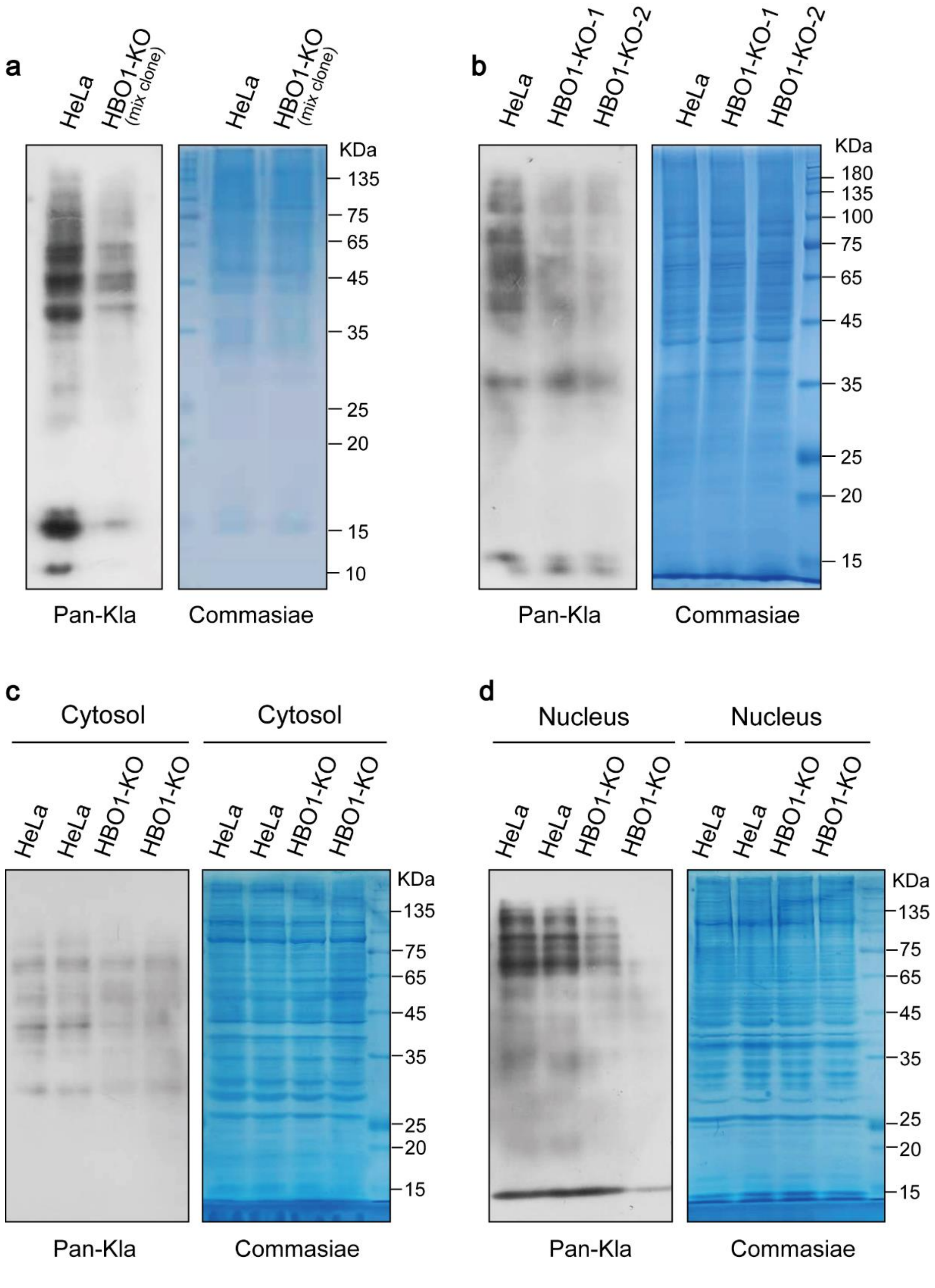


**Supplementary Fig. 4. HBO1 knockout caused down-regulation of lactylation modification level of whole protein in HeLa cells. a-b** HBO1 knockout caused down-regulation of lactylation modification levels of the whole protein in both HBO1 knockout mix clones **(a)** and two clonal cells **(b)**. **c-d** HBO1 knockout caused downregulation of lactylation levels in cytoplasm **(c)** and nucleus **(d)**. HeLa cells were used as control to detect the changes of lactylation level. It should be noted that the lactylation level of the nucleus was more obviously down-regulated than that of the cytoplasm. All immunoblots had three biological repetitions, with similar results. Source data are provided as a Source Data file.

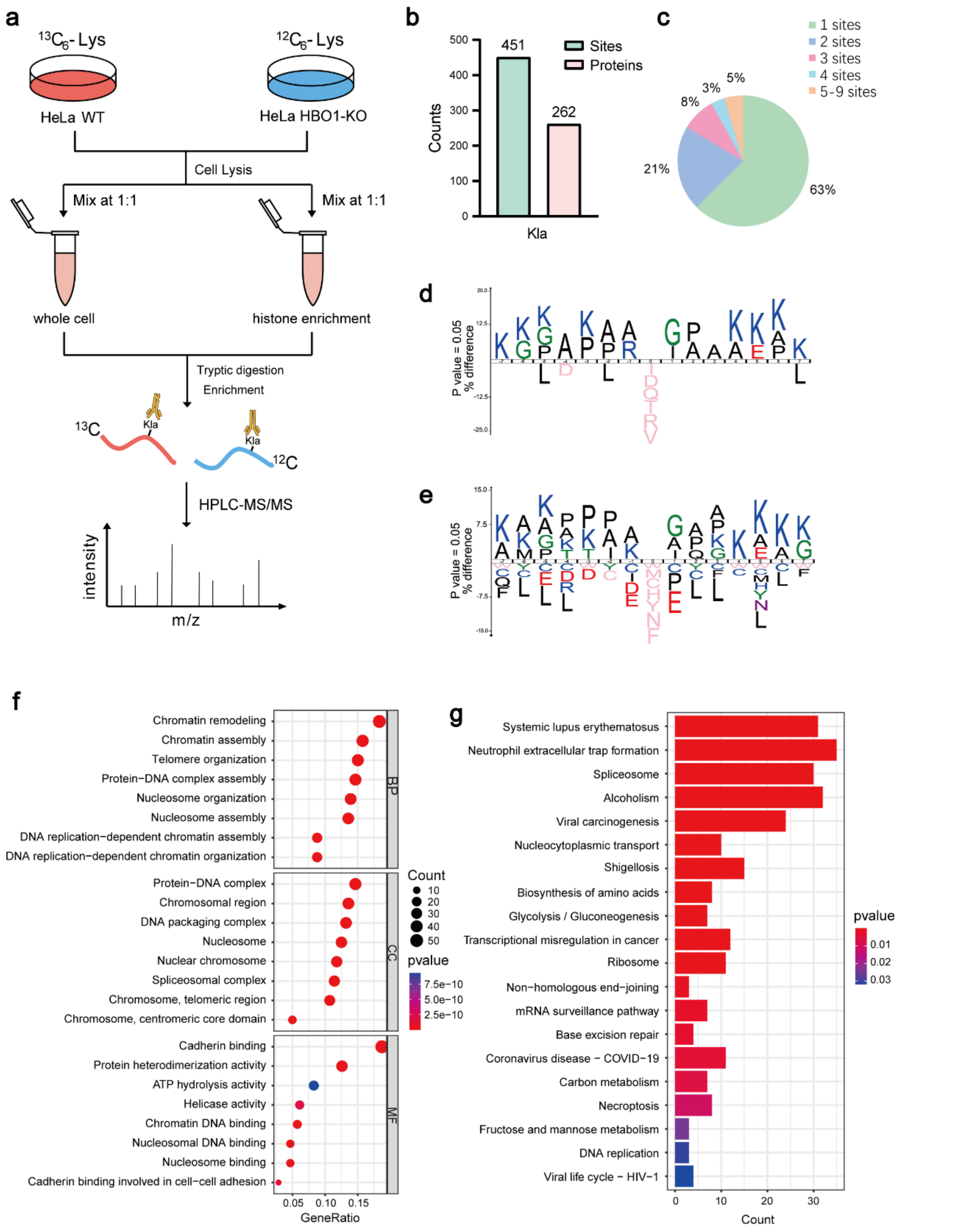


**Supplementary Fig. 5. Characterization of the Kla proteome in HeLa cells. a** Schematic representation of experimental workflow for SILAC quantification. **b** Statistical analysis of Kla protein and sites identified by SILAC in HeLa cells. A total of 451 Kla sites and 262 proteins were identified. **c** The pie chart showed the frequency of Kla occurs on proteins. **d** Sequence motif logo showed a representative sequence for HBO1-regulated Kla sites in HeLa cells. **e** Sequence motif logo showed a representative sequence for all Kla sites identified. **f-g** GO analysis **(e)** and KEGG analysis **(f)** were performed for all Kla proteins identified in HeLa cells. Over-representation test was used to calculated GO and KEGG term enrichment with FDR for multiple test correction. The p-value cutoff = 0.05 and q-value cutoff = 0.1 were selected as the cutoff criteria. Benjamini and Hochberg correction was used to adjust p-values.

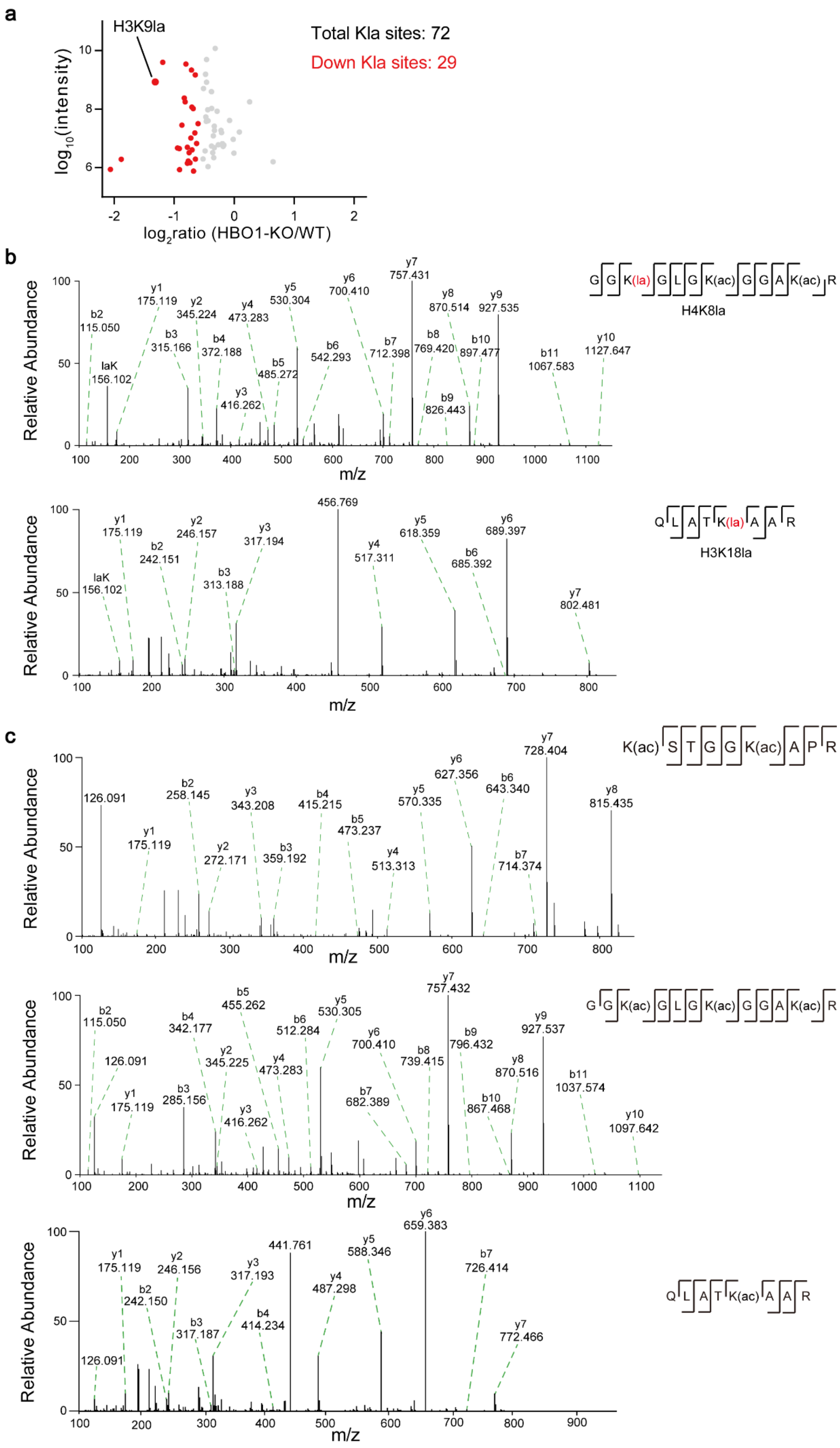
a
H3K9la
Total Kla sites: 72
Down Kla sites: 29
log10(intensity)
log2ratio (HBO1-KO/WT)
b
Relative Abundance
m/z
G G K(la) G L G K(ac) G G A K(ac) R
H4K8la
b2 115.050
laK 156.102
y1 175.119
b3 315.166
y2 345.224
b4 372.188
y3 416.262
y4 473.283
b5 485.272
y5 530.304
b6 542.293
y6 700.410
b7 712.398
y7 757.431
b8 769.420
b9 826.443
y8 870.514
b10 897.477
y9 927.535
b11 1067.583
y10 1127.647
Q L A T K(la) A A R
H3K18la
laK 156.102
y1 175.119
b2 242.151
y2 246.157
b3 313.188
y3 317.194
456.769
y4 517.311
y5 618.359
b6 685.392
y6 689.397
y7 802.481
c
K(ac) S T G G K(ac) A P R
126.091
y1 175.119
b2 258.145
y2 272.171
y3 343.208
b3 359.192
b4 415.215
b5 473.237
y4 513.313
y5 570.335
y6 627.356
b6 643.340
b7 714.374
y7 728.404
y8 815.435
G G K(ac) G L G K(ac) G G A K(ac) R
b2 115.050
126.091
y1 175.119
b3 285.156
b4 342.177
y2 345.225
y3 416.262
b5 455.262
y4 473.283
b6 512.284
y5 530.305
b7 682.389
y6 700.410
b8 739.415
y7 757.432
b9 796.432
b10 867.468
y8 870.516
y9 927.537
b11 1037.574
y10 1097.642
Q L A T K(ac) A A R
126.091
y1 175.119
b2 242.150
y2 246.156
b3 317.187
y3 317.193
b4 414.234
441.761
y4 487.298
y5 588.346
y6 659.383
b7 726.414
y7 772.466

**Supplementary Fig. 6. Characteristics of Kla histone sites regulated by HBO1. a** Scatter plot showed the ratio of histone Kla peptides in HBO1-KO HeLa cells to normal HeLa cells. It is worth noting that H3K9la ratio is lowest in histone H3 and H4. **b** The MS/MS spectra of H4K8la peptide (GGK(la)GLGK(ac)GGAK(ac)R) and H3K18la peptide (QLATK(la)AAR) in the enriched Kla sample of HBO1-KO HeLa cells. **c** The MS/MS spectra of Kac of identical peptides with H3K9la peptide, H4K8la peptide and H3K18la peptide.

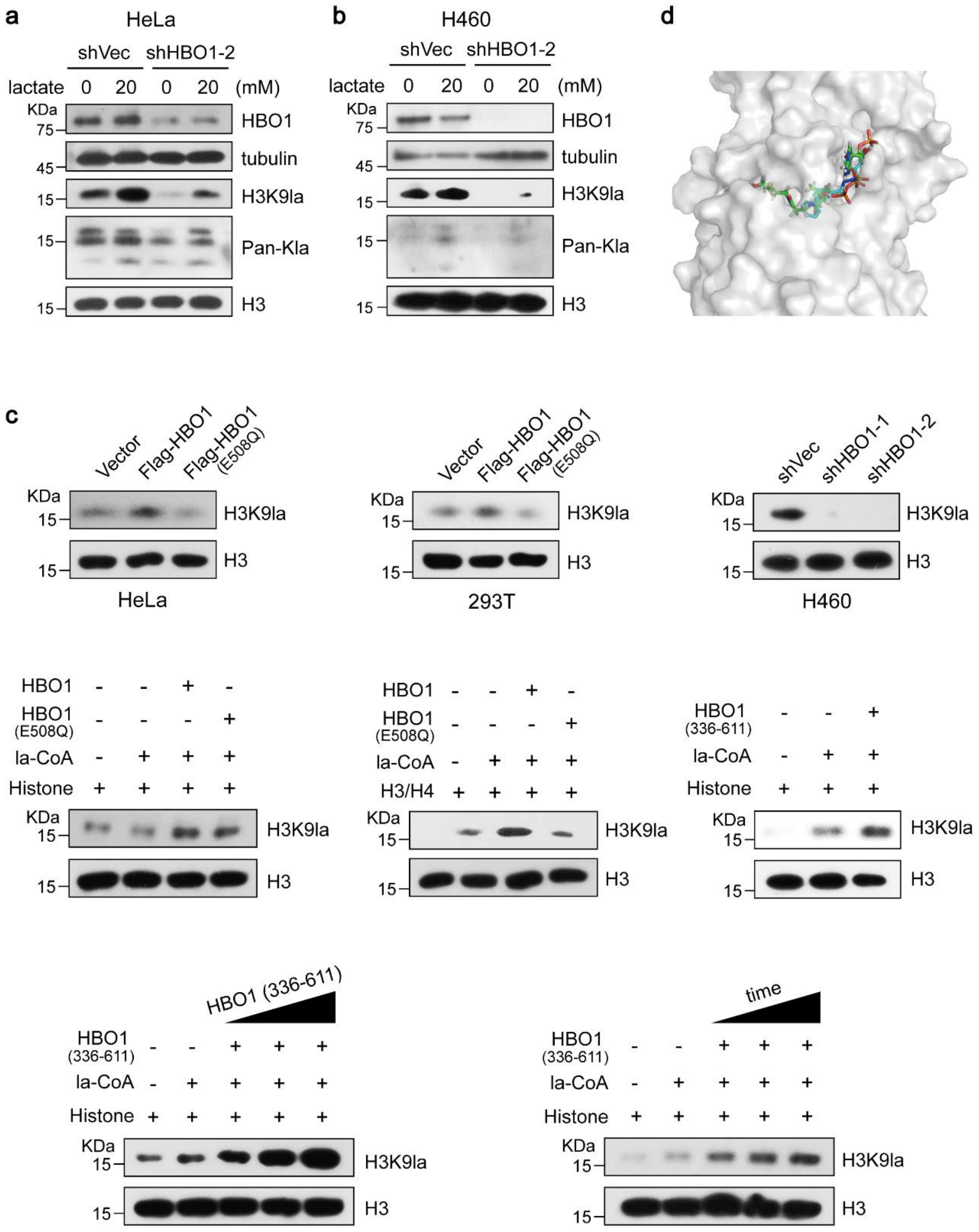


**Supplementary Fig. 7. The HBO1 protein prefers to catalyze the histone H3K9la site. a-b** Lactylation of histones is lactate-dependent mediated by HBO1. Consistent results were obtained for both HeLa cells **(a)** and H460 cells **(b)**. **c** The catalytic activity of HBO1 preference for H3K9la was verified by a series of experiments including HBO1 overexpression and knockdown, as well as enzyme activity in vitro. **d** The space-filling model showed that WM-3835 overlapped with lactyl-CoA, indicating that WM-3835 was bound at the lactyl-CoA binding site of HBO1. All immunoblots had three biological repetitions, with similar results. Source data are provided as a Source Data file.

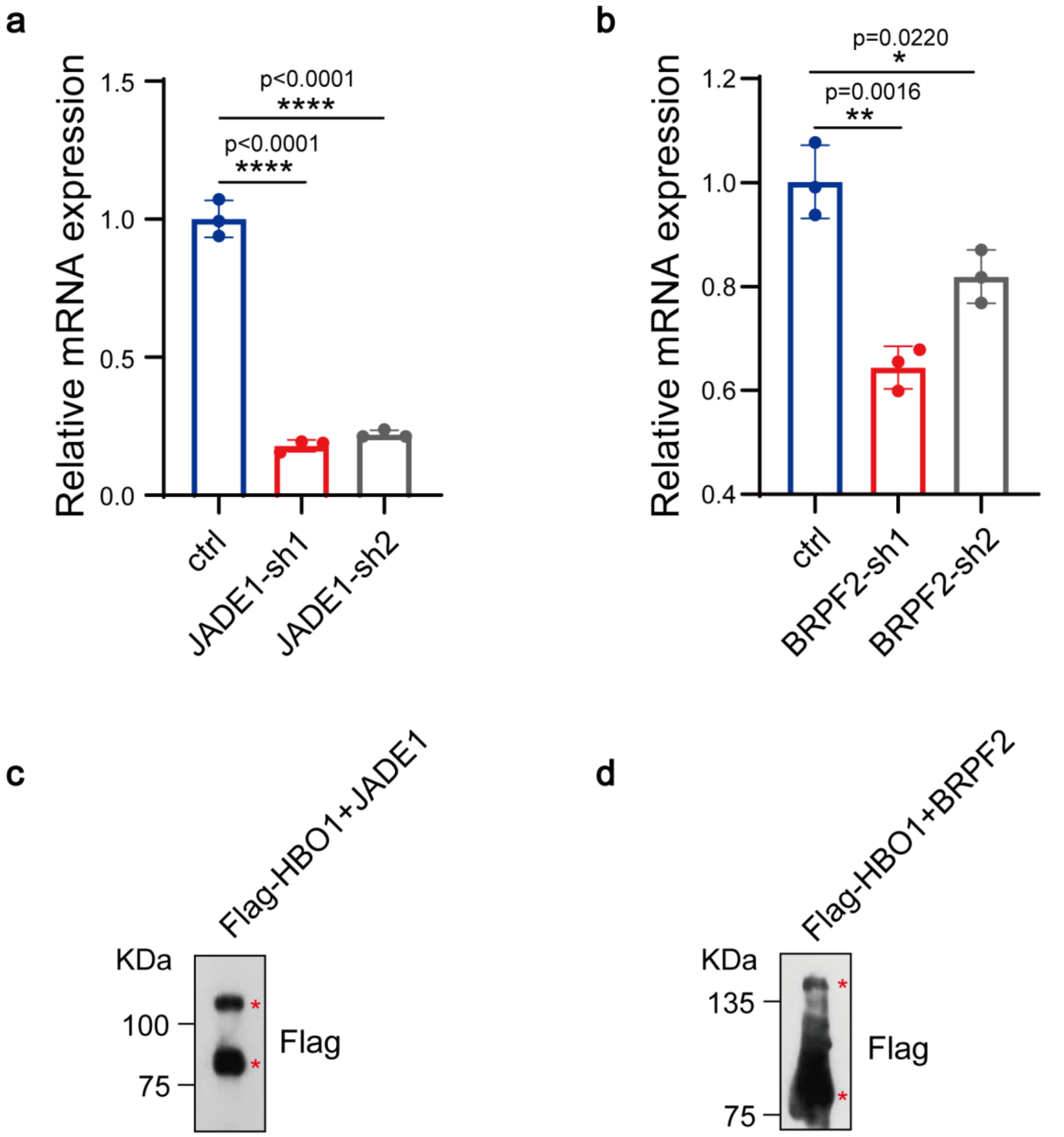


**Supplementary Fig. 8. Identification of scaffold protein expression. a** The bar chart showed the relative mRNA expression level of JADE1 gene after JADE1 knockdown. Data are presented as mean ± SD, two-tailed Student's t-test. P-values are indicated in the figure (n=3 biological repetitions). **c** The bar chart showed the relative mRNA expression level of BRPF2 gene after BRPF2 knockdown. Data are presented as mean ± SD, two-tailed Student's t-test. P-values are indicated in the figure **c-d** Flag-HBO1 and its complex Flag-JADE1 **(c)**, Flag-BRPF2 **(d)** were co-expressed in HEK293T cells, respectively. Purification of the target protein by immunoaffinity. The asterisk (*) indicates the destination strip. All immunoblots had three biological repetitions, with similar results. Source data are provided as a Source Data file.

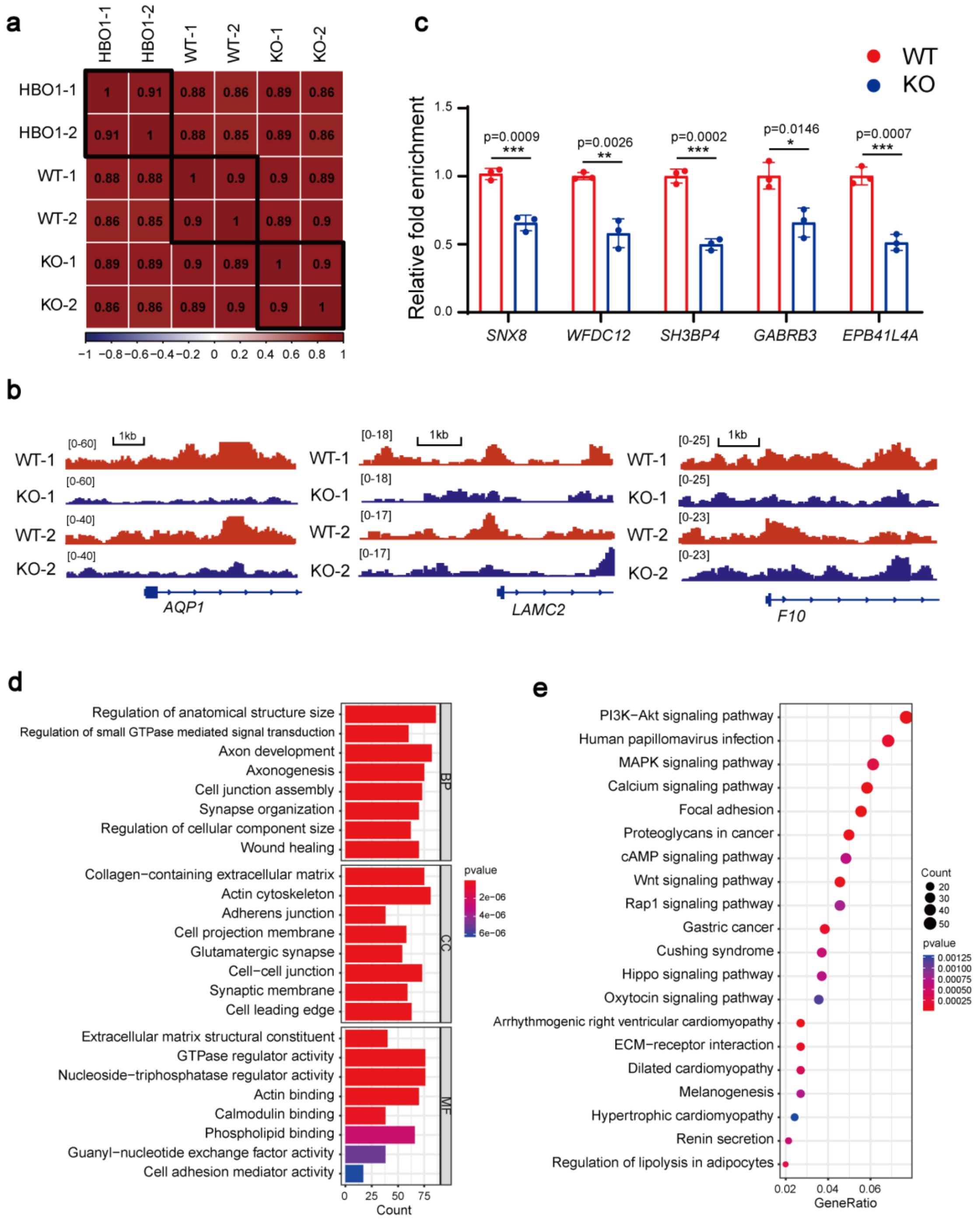


**Supplementary Fig. 9. Genomic analysis of transcriptional consequences of H3K9la. a** Biological duplicates of CUT&Tag showed reproducibility of CUT&Tag assay. Pearson's correlation analysis of two HBO1, H3K9la replicates. Two biological replicates for both WT and HBO1-KO cells showed high consistency of CUT&Tag data. **b** Two representative traces of CUT&Tag showed that H3K9la was enriched in the TSS region of *AQP1*, *LAMC2* and *F10* genes. The red and blue tracks represent the peak of WT and HBO1-KO cells enrichment, respectively. Two biological repetitions, with similar results. **c** CUT&Tag results were verified by qPCR analysis. The results showed level of promoter *SNX8*, *WFDC12*, *SH3BP4*, *GABRB3* and *EPB41L4A* genes enriched with H3K9la in WT and HBO1-KO cells. Data are mean ± SD, two-tailed Student's t-test. P-values are indicated in the figure (n=3 biological repetitions). **d-e** GO analysis **(d)** and KEGG

analysis **(e)** of down-regulated H3K9la binding peaks on candidate target genes. Over-representation test was used to calculated GO term and KEGG enrichment with FDR for multiple test correction. The p-value cutoff = 0.05 and q-value cutoff = 0.2 were selected as the cutoff criteria. Source data are provided as a Source Data file.

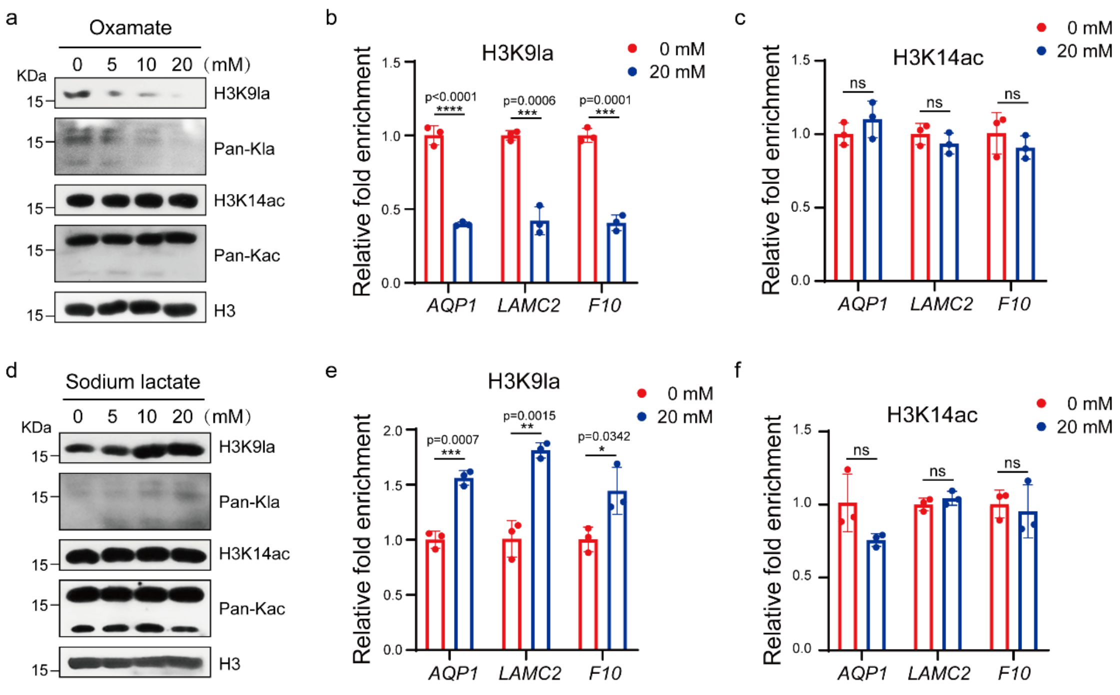


**Supplementary Fig. 10. H3K9la was enriched in TSSs of the gene. a** Western blotting analysis showed that global histone lactate modification and H3K9la modification, but not acetylation and H3K14ac were dose-dependent on Oxamate. The experiment was independently repeated three times with similar results. **b-c** H3K9la was enriched in *AQP1*, *LAMC2*, *F10* promoters. QPCR analysis of H3K9la **(b)** and H3K14ac **(c)** status on the promoter of *AQP1*, *LAMC2* and *F10* after treatment of HeLa cells with 20mM oxamate. **d** Western blotting analysis showed that exogenous sodium lactate increased the levels of global histone Kla and H3K9la but not Kac and H3K14ac in HeLa cells. The experiment was independently repeated three times with similar results. **e-f** H3K9la is enriched in promotors of *AQP1*, *LAMC2* and *F10* promoters. QPCR analysis of H3K9la and H3K14ac states on the promoters of *AQP1*, *LAMC2* and *F10* of HeLa cells treated with 20 mM sodium lactate. Data are presented as mean ± SD, two-tailed Student's t-test. P-values are indicated in the figure (n=3 biological repetitions). Source data are provided as a Source Data file. $p < 0.05$(*), $p < 0.01$ (**), $p < 0.001$ (***), $p < 0.0001$ (****) and ns (no significance).

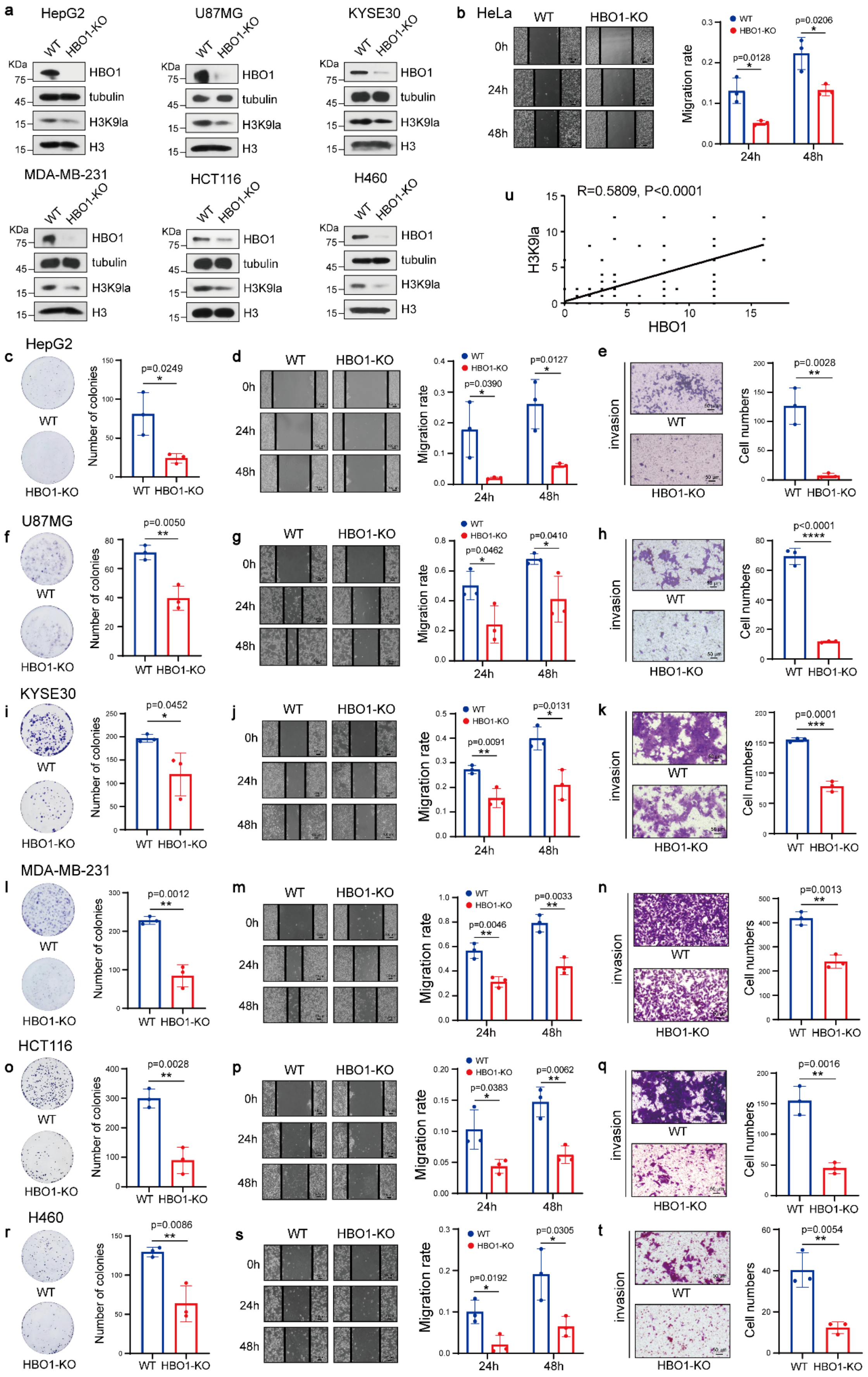

a
HepG2
U87MG
KYSE30
MDA-MB-231
HCT116
H460
WT
HBO1-KO
KDa
75
45
15
HBO1
tubulin
H3K9la
H3
b
HeLa
0h
24h
48h
WT
HBO1-KO
Migration rate
p=0.0128
p=0.0206
u
R=0.5809, P<0.0001
H3K9la
HBO1
c
p=0.0249
Number of colonies
d
p=0.0390
p=0.0127
e
invasion
Cell numbers
p=0.0028
f
p=0.0050
g
p=0.0462
p=0.0410
h
p<0.0001
i
p=0.0452
j
p=0.0091
p=0.0131
k
p=0.0001
l
p=0.0012
m
p=0.0046
p=0.0033
n
p=0.0013
o
p=0.0028
p
p=0.0383
p=0.0062
q
p=0.0016
r
p=0.0086
s
p=0.0192
p=0.0305
t
p=0.0054

**Supplementary Fig. 11. HBO1 knockout caused the downregulation of H3K9la and inhibition of malignant behavior of cancer cells. a** Western blotting analysis showed that HBO1 knockout could induce down-regulation of H3K9la modification levels in HepG2, U87MG, KYSE30, MDA-MB-231, HCT116 and H460 cancer cells. The experiment was independently repeated three times with similar results. **b-t** A series of cell clonal formation experiments, scratch experiments and transwell invasion experiments showed that HeLa, HepG2, U87MG, KYSE30, MDA-MB-231, HCT116 and H460 cells had decreased proliferation **(c,f,i,l,o,r)**, migration **(b,d,g,j,m,p,s)** (Scale, 100 μm) and invasion **(e,h,k,n,q,t)** (Scale, 50 μm) ability after HBO1 knockout (KO) and causing a downregulation of H3K9la. Number of colonies, migration rate and cell numbers were analyzed by Image J software. The bar graphs represent means ± SD (two-tailed Student's t-test) of three independent experiments (n=3). P-values are indicated in the figure. **u** HBO1 expression and H3K9la level were positively correlated with the development of cervical tumors. Spearman correlation analysis of IHC score was performed (R=0.5809). P-values are indicated ($P<0.0001$). Source data are provided as a Source Data file.
