# Supplementary material for: HBO1 catalyzes lysine lactylation and mediates histone H3K9la to regulate gene transcription
Source: Nat Commun. 2024 Apr 26;15:3561. doi: 10.1038/s41467-024-47900-6 (PMC11053077; doi:10.1038/s41467-024-47900-6)
Supplement: Supplementary file 3 — Description of Additional Supplementary Files [file 41467_2024_47900_MOESM3_ESM.pdf]

Description of Additional Supplementary Files

File Name: Supplementary Data 1
Description: The data of ITC experiments.

File Name: Supplementary Data 2
Description: The quantification result of lactylated proteomes with WT HeLa and HBO1-KO HeLa.

File Name: Supplementary Data 3
Description: The quantification result of lactylation of histones WT HeLa and HBO1-KO HeLa.

File Name: Supplementary Data 4
Description: Detailed parameters of HPLC-MS/MS analysis of Kla.

File Name: Supplementary Data 5
Description: The list of antibodies, reagents, and primers used in this paper.
