# Supplementary material for: HBO1 catalyzes lysine lactylation and mediates histone H3K9la to regulate gene transcription
Source: Nat Commun. 2024 Apr 26;15:3561. doi: 10.1038/s41467-024-47900-6 (PMC11053077; doi:10.1038/s41467-024-47900-6)
Supplement: Supplementary file 9 — Reporting Summary [file 41467_2024_47900_MOESM9_ESM.pdf]

Corresponding author(s): Kai Zhang

Last updated by author(s): Apr 13, 2024

Reporting Summary

Nature Portfolio wishes to improve the reproducibility of the work that we publish. This form provides structure for consistency and transparency in reporting. For further information on Nature Portfolio policies, see our Editorial Policies and the Editorial Policy Checklist.

Statistics

For all statistical analyses, confirm that the following items are present in the figure legend, table legend, main text, or Methods section.

n/a	Confirmed	
☐	☒	The exact sample size (*n*) for each experimental group/condition, given as a discrete number and unit of measurement
☐	☒	A statement on whether measurements were taken from distinct samples or whether the same sample was measured repeatedly
☐	☒	The statistical test(s) used AND whether they are one- or two-sided<br>*Only common tests should be described solely by name; describe more complex techniques in the Methods section.*
☒	☐	A description of all covariates tested
☒	☐	A description of any assumptions or corrections, such as tests of normality and adjustment for multiple comparisons
☐	☒	A full description of the statistical parameters including central tendency (e.g. means) or other basic estimates (e.g. regression coefficient) AND variation (e.g. standard deviation) or associated estimates of uncertainty (e.g. confidence intervals)
☐	☒	For null hypothesis testing, the test statistic (e.g. *F*, *t*, *r*) with confidence intervals, effect sizes, degrees of freedom and *P* value noted<br>*Give P values as exact values whenever suitable.*
☒	☐	For Bayesian analysis, information on the choice of priors and Markov chain Monte Carlo settings
☒	☐	For hierarchical and complex designs, identification of the appropriate level for tests and full reporting of outcomes
☐	☒	Estimates of effect sizes (e.g. Cohen's *d*, Pearson's *r*), indicating how they were calculated

Our web collection on statistics for biologists contains articles on many of the points above.

Software and code

Policy information about availability of computer code

Data collection	Protemic mass spectrometry (MS) data was acquired using Xcalibur. link (Thermo Fisher Science). Immunofluorescence staining data was acquired by ZEN 2.3 lite. Isothermal titration calorimetry (ITC) data was obtained by MicroCal PEAQ-ITC isothermal titration calorimeter (Malvern Instruments). Clone formation data were captured by taking pictures with a digital camera. Transwell invasion data and wound healing data were collected through Image View (v.3.7.10565). Immunohistochemistry (IHC) data was acquired by Leica Aperio GT450.
Data analysis	Protemic mass spectrometry raw data were analyzed by MaxQuant (v.1.5.5.1). ITC data were analyzed by Origin (v.8.0, OriginLab). Docking were analyzed by AutoDock Vina (v4.2.6) PyMol (v.3.11.2) and LigPlus (v.2.2). Clone formation data and wound healing data were analyzed by Image J (v.Image J2). CUT&Tag data were analyzed by FastQC version (v0.11.9), Bowtie2 (v2.4.5),Picard tools (version 2.27.4), Samtools version (1.15.1) and deepTools (version 3.5.1). Statistical analysis were analyzed by GraphPad Prism software (version 8.0). Signals of immunohistochemistry (IHC) data were visually quantified using a scoring system from 1 to 3, multiplied intensity of signal and percentage of positive cells

For manuscripts utilizing custom algorithms or software that are central to the research but not yet described in published literature, software must be made available to editors and reviewers. We strongly encourage code deposition in a community repository (e.g. GitHub). See the Nature Portfolio guidelines for submitting code & software for further information.

Data

Policy information about availability of data

All manuscripts must include a data availability statement. This statement should provide the following information, where applicable:

- Accession codes, unique identifiers, or web links for publicly available datasets
- A description of any restrictions on data availability
- For clinical datasets or third party data, please ensure that the statement adheres to our policy

Source data are provided with this paper. The mass spectrometry proteomics data have been deposited to the ProteomeXchange Consortium via the PRIDE partner repository with the dataset identifier PXD051415 [http://www.ebi.ac.uk/pride/archive/projects/PXD051415]. The CUT&Tag data generated in this study have been deposited in the Gene Expression Omnibus (GEO) repository under accession code GSE239656 [https://www.ncbi.nlm.nih.gov/geo/query/acc.cgi?acc=GSE239656]. The raw data generated in this study are provided in the Source Data file. All data needed to evaluate the conclusions in the paper are present in the paper, the Supplementary information and Source Data file.

Research involving human participants, their data, or biological material

Policy information about studies with human participants or human data. See also policy information about sex, gender (identity/presentation), and sexual orientation and race, ethnicity and racism.

Reporting on sex and gender	All the patients of commercial tissue microarray containing 84 cervial cancer and 52 para-tumor tissues are female.
Reporting on race, ethnicity, or other socially relevant groupings	All the patients of commercial tissue microarray containing 84 cervial cancer and 52 para-tumor tissues are Chinese.
Population characteristics	Information not collected. A commercial tissue microarray containing 84 cervial cancer and 52 para-tumor tissues were purchased from Shanghai Zhuoli Biotech company.
Recruitment	We did not recruit any patients in this study. A commercial tissue microarray containing 84 cervial cancer and 52 para-tumor tissues were purchased from Shanghai Zhuoli Biotech company.
Ethics oversight	Cervical tumor tissue microarray ZL-Utrsur1601 was purchased from WELLBI. Our study complies with all relevant ethical regulations and was approved by the Ethics Committee of Shanghai Zhuoli Biotech Company (Shanghai, China) (Ethics number: ZLL-15-01).

Note that full information on the approval of the study protocol must also be provided in the manuscript.

Field-specific reporting

Please select the one below that is the best fit for your research. If you are not sure, read the appropriate sections before making your selection.

☒ Life sciences ☐ Behavioural & social sciences ☐ Ecological, evolutionary & environmental sciences

For a reference copy of the document with all sections, see nature.com/documents/nr-reporting-summary-flat.pdf

Life sciences study design

All studies must disclose on these points even when the disclosure is negative.

Sample size	Samples of 84 cervical cancer cells and 52 normal cervical samples were used. The cell samples were not predetermined, we used HeLa cells, HEK293T cells, HepG2 cells, U87MG cells, MDA-MB-231 cells, KYSE-30 cells, HCT116 cells and H460 cells as study samples, and all the experiments were biologically repeated at least three times.
Data exclusions	No data were excluded.
Replication	All experiments were successfully replicated at least three independent biological experiments, except SILAC MS was performed once and CUT&Tag was performed twice. In the SILAC MS experiment, we strictly controlled the labeling efficiency of isotopes above 98%. In the process of SILAC experiment, heavy and light samples are mixed, digested and identified at the same time, which greatly reduces the errors caused by equipment and experimental operation, thus the results of SILAC experiment have relatively high accuracy. At the same time, we also conducted biological verification of the accuracy of SILAC MS results. Chip-qPCR was performed to guarantee the accuracy of differential gene between WT and HBO1-KO .
Randomization	No randomization was necessary as only single variables changed per experiment. Sex and/or gender were not taken into account in this study.
Blinding	Not applicable. Any phenotypic assessment or other measurements was performed using discrete, quantitative measurements.

Reporting for specific materials, systems and methods

We require information from authors about some types of materials, experimental systems and methods used in many studies. Here, indicate whether each material, system or method listed is relevant to your study. If you are not sure if a list item applies to your research, read the appropriate section before selecting a response.

Materials & experimental systems

n/a	Involved in the study
☐	☒ Antibodies
☐	☒ Eukaryotic cell lines
☒	☐ Palaeontology and archaeology
☒	☐ Animals and other organisms
☒	☐ Clinical data
☒	☐ Dual use research of concern
☒	☐ Plants

Methods

n/a	Involved in the study
☐	☒ ChIP-seq
☒	☐ Flow cytometry
☒	☐ MRI-based neuroimaging

Antibodies

Antibodies used

Following antibodies were used in this study:
Rabbit anti-MYST2 monoclonal antibody (diluted 1:50 CUT&Tag), CST, Cat# 58418, Lot# 1.
Rabbit-anti-MYST2 polyclonal antibody (diluted 1:1000 WB), Protein-tech, Cat# 13751-1-AP, Lot# 00057784.
Mouse anti DDDDK-Tag (Flag) monoclonal antibody (diluted 1:5000 WB), ABclonal, Cat# AE005, Lot# 9200005002.
Mouse anti-Alpha tubulin monoclonal antibody (diluted 1:5000 WB), Yeasen, Cat# 30304ES60, Lot# T1201161.
Mouse anti-His monoclonal antibody (diluted 1:1000 WB), TRANS, Cat# HT501, Lot# 10026545.
Rabbit anti-Histone H3 polyclonal antibody (diluted 1:2000 WB), PTM Biolabs, Cat# PTM-1002, Lot# RL092913.
Rabbit pan anti-L-lactyllysine polyclonal antibody (diluted 1:1000 WB), PTM Biolabs, Cat# PTM-1401RM, Lot# RN011023.
Rabbit pan anti-acetyllysine polyclonal antibody (diluted 1:1000 WB), PTM Biolabs, Cat# PTM-105RM, Lot# RL120732.
Rabbit anti-H3K9la monoclonal antibody (diluted 1:1000 WB, diluted 1:50 CUT&Tag), PTM Biolabs, Cat# PTM-1419RM, Lot# K121902.
Rabbit anti-H3k14la polyclonal antibody (diluted 1:1000 WB), PTM Biolabs, Cat# PTM-1414, Lot# ZCD018K708P2.
Rabbit anti-H3k18la polyclonal antibody (diluted 1:1000 WB), PTM Biolabs, Cat# PTM-1406, Lot# L031701.
Rabbit anti-H4K5la monoclonal antibody (diluted 1:1000 WB), PTM Biolabs, Cat# PTM-1407RM, Lot# L020709.
Rabbit anti-H4K8la monoclonal antibody (diluted 1:1000 WB), PTM Biolab, Cat# PTM-1415RM, Lot# K121904.
Rabbit anti-H4K12la monoclonal antibody (diluted 1:1000 WB), PTM Biolab, Cat# PTM-1411RM, Lot# K122425.
Rabbit anti-H4K16la monoclonal antibody (diluted 1:1000 WB), PTM Biolab, Cat# PTM-1417RM, Lot# K120927.
Rabbit anti-H3k14ac monoclonal antibody (diluted 1:2000 WB), PTM Biolab, Cat# PTM-113RM, Lot# RM050606.
Anti-L-lacytyllysine antibody conjugated agarose beads (diluted 1:1000 IP), PTM Biolabs, Cat# PTM-1404, Lot# TAL301B01.
Rabbit anti-BRPF2 polyclonal antibody (diluted 1:1000 WB), ABclonal, Cat# A9869, Lot# 0086060101.
Rabbit anti-JADE1 polyclonal antibody (diluted 1:1000 WB), Protein-tech, Cat# 28472-1-AP, Lot# 00092496.
Horseradicase-tagged Rabbit Anti-Goat IgG (H+L) (diluted 1:10000 WB), ZSGB-BIO, Cat# ZB-2301, Lot# 232251022.
Horseradicase-tagged Rabbit Anti-Goat IgG (H+L) (diluted 1:10000 WB), ZSGB-BIO, Cat# ZB-2305, Lot# 2356807114.
Alexa Fluor 647-labeled Goat anti-Rabbit IgG (H+L) (diluted 1:100 IF), Beyotime, Cat# A0468, Lot# 090619200821.
FITC-labeled Goat anti-Mouse IgG (H+L) (diluted 1:100 IF), Beyotime, Cat# A0568, Lot# 032420200803.

Validation

All antibodies we used were validated by manufacturers and the validation informations were provided on their websites. Moreover, MYST2 (CST, and Protein-tech), Pan-Kac, and H3K14ac antibodies were exactly validated (Wong J et al, Nucleic Acids Res, 2021). Also, the Pan-Kla, H3K9la, H3K18la, H4K8la and H3 antibodies have been shown to have high specificity (Zhao Y et al, Nature, 2019).
Manufacturer validation:
Anti-MYST2 CST (Cat# 58418, Lot# 1)
Species: all; Application: WB, IP, IHC, CHIP, C&R; Manufacturer's web site:https://www.cellsignal.cn/products/primary-antibodies/myst2-d4n3f-rabbit-mab/
Anti-MYST2 Protein-tech (Cat# 13751-1-AP, Lot# 00057784)
Species: all; Application: IF, IHC, IP, WB, ELISA; Manufacturer's web site:https://abclonal.com.cn/catalog/AE005
Anti DDDDK-Tag mAb Protein-ABclonal (Cat# AE005, Lot# 9200005002)
Species: all; Application: IF, IHC, IP, WB, ELISA; Manufacturer's web site:https://ptgcn.com/products/Flag-tag-Antibody-66008-4-Ig.htm
Anti-Alpha tubulin Yeasen (Cat# 30304ES60, Lot# T1201161)
Species: all; Application: IF, IHC, IP, WB; Manufacturer's web site:https://www.yeasen.com/products/detail/887
Anti-His-tag TRANS (Cat# HT501, Lot# 10026545)
Species: all; Application:WB; Manufacturer's web site:https://www.transgen.com/antibody_tag/385.html
Anti-Histone H3 PTM Biolabs (Cat# PTM-1002, Lot# RL092913)
Species: all; Application:WB, IHC; Manufacturer's web site:http://www.ptm-biolab.com.cn/productDetail.html?id=4601
Pan anti-Kla PTM Biolabs (Cat# PTM-1401RM, Lot# RN011023)
Species: all; Application:WB, ICC, FC, CHIP, IP, IHC; Manufacturer's web site:http://www.ptm-biolab.com.cn/productDetail.html?id=5863

Pan anti-Kac PTM Biolabs (Cat# PTM-105, Lot# 12839112M530)
Species: all; Application:WB, CHIP, IP, IHC; Manufacturer’s web site:http://www.ptm-biolab.com.cn/productDetail.html?id=6244
Anti-H3K9la PTM Biolabs (Cat# PTM-1419RM, Lot# K121902)
Species: all; Application:WB, CHIP, ICC/IF, IHC; Manufacturer’s web site:http://www.ptm-biolab.com.cn/productDetail.html?id=5381
Anti-H3k14la PTM Biolabs (Cat# PTM-1414, Lot# ZCD018K708P2)
Species: all; Application:WB, CHIP; Manufacturer’s web site:http://www.ptm-biolab.com.cn/productDetail.html?id=5196
Anti-H3k18la PTM Biolabs (Cat# PTM-1406, Lot# L031701)
Species: all; Application:WB, CHIP, IHC, ICC/IF; Manufacturer’s web site:http://www.ptm-biolab.com.cn/productDetail.html?id=5558
Anti-H4K5la PTM Biolabs (Cat# PTM-1407RM, Lot# L020709)
Species: all; Application:WB, IP, FC, IHC, ICC/IF; Manufacturer’s web site:http://www.ptm-biolab.com.cn/productDetail.html?id=5191
Anti-H4K8la PTM Biolabs (Cat# PTM-1415RM, Lot# K121904)
Species: all; Application:WB, CHIP, C&R; Manufacturer’s web site:http://www.ptm-biolab.com.cn/productDetail.html?id=5252
Anti-H4K12la PTM Biolabs (Cat# PTM-1411RM, Lot# K122425)
Species: all; Application:WB, CHIP, IHC, IP; Manufacturer’s web site:http://www.ptm-biolab.com.cn/productDetail.html?id=5251
Anti-H4K16la PTM Biolabs (Cat# PTM-1417RM, Lot# K120927)
Species: all; Application:WB, CHIP, IHC, ICC/IF; Manufacturer’s web site:http://www.ptm-biolab.com.cn/productDetail.html?id=5199
Anti-H3K14ac PTM Biolabs (Cat# PTM-113RM, Lot# RM050606)
Species: all; Application:WB, CHIP, IHC, ICC/IF; Manufacturer’s web site:http://www.ptm-biolab.com.cn/productDetail.html?id=5555
Anti-L-lacytyllysine antibody conjugated agarose beads PTM Biolabs (Cat# PTM-1404, Lot# TAL301B01)
Species: all; Application:IP; Manufacturer’s web site:http://www.ptm-biolab.com.cn/genericAntibody.html?id=4725
Anti-BRPF2 ABclonal (Cat# A9869, Lot# 0086060101)
Species: all; Application:WB; Manufacturer’s web site:https://abclonal.com.cn/catalog/A9869
Anti-JADE1 Protein-tech (Cat# 28472-1-AP, Lot# 00092496)
Species: all; Application:WB, IF, ELISA; Manufacturer’s web site:https://www.ptgcn.com/products/PHF17-Antibody-28472-1-AP.htm
Alexa Fluor 647-labeled Goat anti-Rabbit IgG (H+L), Beyotime (Cat# A0468, Lot# 090619200821)
Species: all; Application:IF; Manufacturer’s web site:https://www.beyotime.com/product/A0468.htm
FITC-labeled Goat anti-Mouse IgG (H+L), Beyotime (Cat# A0568, Lot# 032420200803)
Species: all; Application:IF; Manufacturer’s web site:https://www.beyotime.com/product/A0568.htm
Horseradicase-tagged Rabbit Anti-Goat IgG (H+L), ZSGB-BIO, (Cat# ZB-2301, Lot# 232251022)
Species: all; Application:WB; Manufacturer’s web site:http://www.zsbio.com/
Horseradicase-tagged Mouse Anti-Goat IgG (H+L), ZSGB-BIO, (Cat# ZB-2305, Lot# 235680714)
Species: all; Application:WB; Manufacturer’s web site:http://www.zsbio.com/

Eukaryotic cell lines

Policy information about cell lines and Sex and Gender in Research

Cell line source(s)	HeLa cells, HEK293T cells, HepG2 cells, U87MG cells, MDA-MB-231 cells, KYSE30 cells,HCT116 cells, H460 cells. These cell lines were purchased from Procell Life Science&Technology Co.,Ltd.
Authentication	No further authentication of cell line was performed.
Mycoplasma contamination	Mycoplasma contamination was detected by cytoplasmic DAPI staining, and no contamination during experiments.
Commonly misidentified lines (See ICLAC register)	No commonly misidentified cell lines were used.

Plants

Seed stocks	none
Novel plant genotypes	none
Authentication	none

ChIP-seq

Data deposition

- [x] Confirm that both raw and final processed data have been deposited in a public database such as GEO.
- [x] Confirm that you have deposited or provided access to graph files (e.g. BED files) for the called peaks.

Data access links<br>*May remain private before publication.*	https://www.ncbi.nlm.nih.gov/geo/query/acc.cgi?acc=GSE239656
Files in database submission	.bigWig and .fastq files are provided.
Genome browser session<br>(e.g. UCSC)	We only deposit data on GEO and not at the Genome browser. Signal tracks were visualizes using Integrative Genomics Viewer (version 2.15.1).

Methodology

Replicates	The CUT&Tag data were bioreplicated twice.
Sequencing depth	The sequencing depth of each experiment was 6G, and All datasets were sequenced with 2*150 bp paired-end reads.
Antibodies	Anti-HBO1 (CST, Cat# 58418), anti-H3K9la (PTM Biolabs, Cat# PTM-1419RM)
Peak calling parameters	Peaks were identified for each sample and biological replicate using MACS2 (version 2.2.7.1) with command line options "macs2 callpeak -q 0.05 -g hs -f BAM --nomodel"
Data quality	Sequencing quality was evaluated by FastQC version (v0.11.9). All reads were aligned to the human genome build hg38 using the Bowtie2 (v2.4.5) with the default parameters. MACS2 (version 2.2.7.1). was used to identify peaks of enrichment with a FDR cutoff of 0.01.
Software	FastQC version (v0.11.9); Picard tools (version 2.27.4); Bowtie2 version (v2.4.5); Samtools version (1.15.1); deepTools (version 3.5.1); MACS2 (version 2.2.7.1).
